# Appendix

**Published:** 1838-01-01

**Authors:** 


### APPENDIX.

### Westminster Hospital.

### On a peculiar and undescribed Injury of the Shoulder, being the Substance of a Clinical Lecture delivered December 2, by Mr. Guthrie.

I have promised for the last two months to describe to you the nature of this accident, as far as circumstances would permit; but have not been able to obtain the attendance of the individual to whom it happened until this day, and it is of little use describing these things unless you can at the time test the value of the description.

J. Cadman, who is now seated before you, is a plasterer by trade, and when commencing his daily work felt the ladder turn on which he stood, and after some effort to save himself, he fell with it, his left elbow striking the ground, whilst his shoulder rested against one of the steps of the ladder, in a way he cannot distinctly explain. He felt he had sustained a severe injury in the shoulder, and the elbow was much grazed. He was brought immediately to the hospital; but there was so much swelling that the house-surgeon, Mr. Dasent, could not make out the nature of the injury, and sent to me. I saw him about three hours after the accident; and the most remarkable and striking appearance was a fold or pucker of the skin, the size of the half of half-a-crown, situated over the middle of the pectoral muscle, where it forms the anterior fold of the arm-pit. A hard substance could be felt below this, and extending above it towards the coracoid process, which could not be distinguished on account of the swelling, and it had been supposed that this hard substance was the coracoid process broken off. The head of the humerus could be very distinctly felt on the outer part of the glenoid cavity, or something like it. The arm was very moveable in every direction, and the elbow could be brought close to the side, and made to strike the ribs without difficulty. I decided that it was a fracture, and not a dislocation; but the nature of the fracture I did not understand, and hoped it would become apparent when the swelling had gone down. The fore-arm was bent, the arm brought close to the side on a splint, and leeches and cold lotions were applied and repeated until the swelling very slowly subsided. I was now satisfied that the humerus had been broken at its anatomical neck, and forced through the pectoral muscle, the fascia covering which and the skin had offered sufficient resistance to prevent its passing through them, and forming a compound fracture; causing the bone, however, to pass upwards, and puckering the skin by carrying it along with it. The arm was shorter, and the retraction of the pectoralis major, and probably of the subscapularis, had drawn the bone more into the situation the head of the humerus usually occupies when dislocated under the pectoral muscle. The shape of the broken end of the bone was satisfactory as to its being a broken bone; but I was not at all pleased with its situation, and as no common ordinary extension moved it downwards, I caused him to be largely bled, and gave him tartar emetic at different doses to 12 grains during an hour and a quarter, that I placed him under a gentle but gradually increasing extension in the pullies. I found I could bring the bone down to its natural situation as to length, but I could not make it remain in its proper place. There was, therefore, nothing to be done but to allow Nature to work for herself; and she has certainly worked wonders, for at this moment Cadman suffers from one inconvenience only, and that is that he cannot touch the ceiling with the hand of the injured side, at the same distance that he can with the other; he is obliged to be five or six inches nearer to it, from the arm making a greater angle with the head, than on the sound side. In all other respects, he can do his work just as well as before. When he sits on a chair, as he now does, with the fore-arm bent resting on the thigh, the hand supine, the prominence of the broken bone is very remarkable, and on placing a dry bone by the side of it, it appears to correspond to the small tubercle on the inside, and to a part of the great tubercle or tuberosity on the outside. The hollow between seems to be that for the passage of the long head of the biceps; but whether this tendon runs in it I cannot ascertain. The tendon of the subscapularis appears to be attached to the inner and back part of the small tuberosity, and to have drawn it inwards, whilst that of the pectoralis major, which is well defined, has drawn it inwards and forwards. The portion of the head of the bone, or the whole perhaps of the cartilaginous surface, remains attached in situ with a part of the great tuberosity; but how far, or how much of the three muscles inserted into this process remain with it and the head, I cannot ascertain. I should think but little of the teres minor. The arm moves with perfect ease in every direction; when it is rotated outwards and backwards, the broken end of the humerus seems as if it were going to come through the skin, it is so prominent, and when the arm is raised as high as it can be done, the prominence of bone is seen above the shoulder, as it then rides as high as the clavicle. I have offered him £50, which he may leave by will, for the dissection of his arm, if he dies before me; or my son will give it to his heirs, if he survives me, as I should like the accident to be fully understood. I have mentioned it to my colleagues of the Court of Examiners, but it is not known to any of them. Sir A. Cooper has sent me the 21st plate of the last edition of his work on fractures and dislocations; but this only shews a fracture of the anatomical neck, with little separation, and that outwardly. Mr. White thinks he has seen a case somewhat like it, from there having been the same sort of pucker in the skin in front; and Mr. Cusack, of Dublin, thinks he has met with one of the same kind, from having also seen the same pucker. Both these cases did well, but with unseemly shoulders. I am of opinion that the elbow came first to the ground, but that the step of the ladder struck almost simultaneously against the head, or rather across the neck of the bone, and that the effect of the first blow, which would have caused a dislocation, was thus modified, and gave rise to a fracture. It is not, after all, of any consequence to know how the thing happened; but it is of importance to know that if nothing is done, Nature will right herself so as to recover the use of the arm. Surgery is not, however, satisfied with this; and my object in a future case of the kind, now that I think I understand it, would be to prevent the deformity, which in a woman's arm would be considerable, although much less than I expected; for the pucker has disappeared, and the humerus under use has resumed so much of its natural direction, that I should never have thought of extension by the pullies if it had always been the same. The pointed ends of the fracture will yet round off, and form a small round extremity of bone, and a kind of false joint with the parts around. There is I presume some ligamentous union with the head of the humerus.

In a case of this kind, I should make extension until the bone resumed its proper place; but this must be done very carefully, for I am not sure it could be done effectively without tearing the skin of the pucker or fold I have described, certainly not without great risk of doing it, and which would render the accident a very dangerous one. If the bone could be brought into its proper place (of which, from this and other causes, I have some doubt), it is probable it would not be easily retained by padding the axilla, and other means which will at the time suggest themselves to you; and if it were, it is possible that bony union in such a situation might be more detrimental to the free use of the arm than the mode of cure which Nature has adopted. I am inclined to believe that the capsular ligament of the joint was not torn, or extensively so; but this must be matter of conjecture.

Having thus drawn your attention to this case, I shall hope it will soon be thoroughly understood; and in order that no misunderstanding may take place on the subject, I shall write, and leave the observations I wish you to remember with the house-surgeon, and all that please may take a copy of them; and I shall adopt this course in future with any thing I may say which I think it useful for you to recollect, or to be able to refer to.

---

The poor blind deaf man, with half-a-dozen shot holes in his body, I now place before you, was once a gay and gallant soldier; he received these wounds on the heights of Roliça, on the 17th August, 1808; and I bring him forward as an instance of the British surgery of the day, in contradistinction to what often followed, and as a proof of that which ought to form our rule of practice for the future in the inquiries, I am this day going to notice, viz. those of the arm from musket-shot.

When I revised and completed my work on gun-shot wounds in the Winter of 1814, I had the support of all the junior medical officers of the Peninsular army, the approbation of all the seniors under whom I had served, except the Army Medical Board (I was too young for them), and the esteem and recommendation of most of my equals. I did not like to say I had seen more than many of them; I had no desire to say so: I was more willing and more happy to divide any credit which might be awarded with my brethren, and to take the responsibility of defending what was objected to on myself, than to withdraw from the strictures, and many of them were sharp ones, which were made in the surgery of the campaign of Waterloo, by saying that it was not the same with the surgery of the Peninsula. You will naturally ask me why it was not? and the answer is a very simple one: that they were not the same people, or when they were the same, the ablest had marched with the army to Paris, or were solely engaged with the wounded officers. The hospitals were principally in charge of others who had served but little on the field of battle, and it was the wounded under their care that were open to remark. One instance will suffice to explain my meaning. After the last battle in the Peninsula, no one on the third day could have found a gun-shot fracture of the thigh in the bent position on the side; after the battle of Waterloo no person could have found one in any other. Here is what was a thigh-bone at the battle of Albuhera; the man died at Elvas, and it looks something like a ram's horn. He lay on his back, and the thigh on its side. And when this is the case, the thigh is always crooked, and the man generally dies. Twenty-two years have passed away since the battle of Waterloo; I have no surgical contemporaries of my own standing in London, and I may now tell you the truth without the fear of giving offence, or of appearing egotistical. You will again ask me perhaps how it was possible that in one short year an error of this kind could have been committed? and why many surgeons preferred serving at hospital stations to the field of battle? and I will have the pleasure of telling you for the benefit of those who may be aspirants for military medical honors.

The first appointment a young man receives at 22 years of age, is, that of an hospital assistant, in which situation on service he is worse treated than any costermonger's donkey in Westminster or Shoreditch; for the donkey is occasionally fed, cleaned, and lodged, but the doctor, if he wishes any of these conveniences of life, must find them for himself wherever he can catch them, and that is not always so easy for a man to do, who has no money in his pockets.

Suppose him, if you please, at Portsmouth, waiting for a fair wind, and spending at the Crown or the Blue Posts the last pound which remains of his two months' pay in advance, which have been very improvidently given him; or land him, if you please, at Lisbon or at St. Andero, with five pounds in his purse, a foretaste of his prudence. He presents himself to the inspector of hospitals, who very politely informs him that he must march to join the Army in three days, and advises him to buy a mule to carry him and his luggage, and to hire a servant, for whom five shillings a week will be paid at a future time. He then furnishes him with an order on the quarter-master-general's office for a route and on the commissary for his rations.

The Quarter-master General very gravely gives him a route, and the Commissary-general very agreeably provides him, on his receipt, with as much meat, bread, and wine, as ought to last him three days on the road, and adds very graciously so much wood to cook the meat; but which it may be supposed the young doctor very obligingly leaves behind him for the next gentleman in a similar situation, and who may be equally unable to carry it. Having no money to buy a mule or a jackass, he sends his trunks to the Stores, where they are soon very cleverly plundered of every thing valuable, and starts with a small sack on his back, containing a clean shirt, and a new pair of shoes. If he should have a little money in his pocket, he ventures to hire some lad who offers himself at the corner of the street, or who is recommended by some person about the house where he lodges, and who, in all probability, very civilly and quietly walks off on the night of the second or third day's march, with the sack and every thing else he can lay his hands upon. If my friend has had the good fortune to attract the attention of the inspector, he will perhaps direct that he be attached to a party of bullock cars or mules going up to the Army with stores, and if this should happen, he will have a chance of saving his baggage and of getting something to eat, but bullock cars travel only two miles an hour on level ground, one on a bad road, and oftentimes wait for an hour to take breath, so that having ten or twelve miles to travel, he is out for twelve or fourteen hours under a burning sun, or in a heavy rain. If he escapes after ten or twenty days of this work, it is only on his arrival at his station to set off back again on a similar travel, or to take charge of a large number of sick, and share the dangers of a crowded hospital. The cemetery called English at Ciudad Rodrigo, contains all that remains of twenty or thirty-one of these gentlemen, the victims of distress and disease. I remember one of these young men at Puebla de la Calçada, a village on the plain of the Guadiana, not far from Merida, who had just come up from Lisbon; the village was full of troops, and as the rank of a hospital mate is the lowest of commissioned officers, his lodgings were none of the best, his bed being on the ground-floor, at an equal distance between the peasant and his wife, and an old sow and a dozen of pigs that had grown up to the size of young porkers able to provide for themselves. From these he was separated by a partition having a door-way, opposite the street entrance, the lower third of which was blocked up by a board, in order to prevent the pigs walking into the room at pleasure. The doctor finding his position in the night rather hot, being in the month of September, shifted his palliass between the doors for the benefit of the air, which came in under the street door. The peasant, who rose at the dawn of day, woke him, and having opened the front door, made signs to him to rise. The doctor was indignant at being thus disturbed out of a sound sleep, and signified that he would not get up. The peasant in his turn was more vociferous and urgent with tongue and signs that he should shift his position; he looked, as the doctor afterwards said, like a talking sign-post. The matter was however soon adjusted, a horn was heard to sound, the peasant tore his hair in despair, out jumped the lady pig right on the back of the sleeper, and then sprung out of the door, followed by all her family, to join the swine-herd who was thus collecting them according to ancient custom, at the end of the village, for their day's pasture in the adjoining fields and on the bank of the river. The roaring of the doctor, and the cries of the peasant's wife, brought in some of the neighbours and soldiers who were also up, and the poor doctor, on being raised, was found to have suffered from only a few bruises. He was however a doomed man; he was only about five feet four high, rather good-looking, but like many other people, had remarkably short thighs and legs. In the evening at sun-set, and the evenings in Autumn in Estremadura after a sultry day are delicious, he thought he would stand at the door, and catch the soft but cool breeze that is always felt at that hour. He was thinking of home, and what could have induced him to leave it, for it was just the hour at which he used to steal from the shop where he was apprenticed in Old Gravel Lane, and take a quiet walk dawn to the bank of the Thames, to enjoy the evening breeze, and study the muscles on the naked men, who appeared like so many demons emptying coals out of the colliers into the coal-barges. His eyes rested on the fine blue sky, so common in Spain, and so rarely seen in this climate, and he almost thought he yet could be happy for a few months in it. At this moment again he heard the sound of the swine-herd horn; it reminded him of the irruption of the morning, and having most emphatically, with a long-drawn sigh, damned those pigs, he continued to meditate and to admire until his attention was drawn to sublunary things by a noise which was, alas! too late. On looking down he saw the old lady pig, followed by all her family, coming right at him, full tilt, accompanied by all the neighbouring pigs who lived beyond him. In an instant she was between his legs. Only conceive my little doctor, with an old sow near six feet long by two feet wide, in such a position—his fate was as inevitable as your's would be in a similar situation;—over he went, bumped his nose against her tail, and rolled covered with blood under the rest of the family, who bolted over him into the stye. He was not aware, poor fellow, that the swine-herd dismissed his flock by sound of horn as well as collected them, and every pig knows that his master or mistress had prepared for him a trough well filled with peas, of which the first comer had the best share. The pigs, on their arrival at the end of the village, awaited most patiently the swine-herd's will; but the moment the first sound of the horn was drawn, every pig took to his heels, and woe to any one who stood in their way. The proximity of their dormitories to their masters and mistresses rendered them perfectly well acquainted; and on their way home, if they met their mistresses, who generally attended to their food, they would jump upon them like so many puppy-dogs. When they got home, if the door was not open, the first arrival jumped up and pulled the string of the latch, and let himself and the rest in; there being no locks nor keys in that primitive country.

Circumstances and accidents, such as I have related, rendered it very difficult however to procure good qualified surgeons for the army, and a curious expedient was resorted to in consequence. Political economists say, that when the demand for tea, coffee, sugar, cotton, &c. &c. is greater than the supply, the price will increase, and that human labour ought to be regulated by the same rule; the authorities in England thought however otherwise, and instead of increasing the price, or smoothing away the difficulties, they deteriorated the article, and as surgeons could not be found qualified to kill or cure by commission, they thought it right to take those of an inferior description, and give them only a warrant, as they do to boatswains and gunners on board-ship. I do not know whether this bright idea originated with the Government or the Army Medical Board; but when elicited, it seemed to please all parties, and a certain number of gentlemen thus warranted to do business came out to Spain. I had some of them at San Andero, where I was for a couple of months. One poor fellow having called to report his arrival, I desired him to sign a paper descriptive of qualifications. This I found he could scarcely do, the letters very much resembling pot-hooks; which he attributed to sea-sickness, in which I sympathized with him, my own stomach being easily unshipped by a very moderate view of blue salt water. I therefore sent him to live at one of the fever-hospitals, to do duty for a few days, until I might see what he was made of. Two days after going to this hospital at six in the morning, I found him in a little alarm. A soldier had been brought to him, he being the officer on duty for the night, riotously drunk; and in order to keep him quiet, he had him tied hand and foot to the four corners of the first bedstead he could meet with, and having thus got the man on his back, recollected he was warranted to do something in physic, he therefore gave him a good dose of tartar-emetic, believing his stomach ought to be emptied; but never thinking, or not knowing that a man could not advantageously vomit on his back, he was found suffocated two or three hours afterwards. I met my friend some three months after this in charge of 40 men and officers at Renteria, going to Passages ill with fever, of which disease he knew nothing; he assured me he had been more fortunate than when with me. He had been sent in charge of sick to several places; on the present occasion he had lost but two; on a former, at Pampeluna three, at Vittoria five; that he hoped to do better in time, and I have little doubt, that if his exploits had been as duly recorded as those of Don Juan, he might have boasted by the end of the campaign of "*tres cientos in Hispanha.*"

Perhaps, gentlemen, you may think those doctors who were higher in rank fared better. I will give you an example in a bit of my own history. Having nearly lost my life through fever, I was sent to England, and returned just at the time Marshal Massena broke up from before the lines and retreated. I bought a horse and a mule, hired a servant on the doubtful recommendation of being as good as any one I could get, and set off to follow the army to Coimbra. On the fourth day at Rio Mayor, the fellow finding I kept too sharp a watch to permit him to rob me of all I had, ran away with one of my two blankets and my dinner. I was now in a happy state, with a horse and a mule to clean, and nothing to eat. An assistant-surgeon, who happened to be travelling the same road, and had joined me the same day, which in all probability, prevented my losing my horse and mule, had a soldier-servant and his mule in the same stable with mine, and he kindly allowed him to see that they were not stolen. The country was desolate; dead horses, asses, and men lay about in all directions, and there was little to be obtained of any kind. My assistant-surgeon, whose name I very ungratefully forget, also presented me with a blanket he said he could spare. It was almost as fatal a gift as the shirt of Nessus. From that time I ceased to sleep, my flesh seemed to be creeping and crawling all night, I became spotted all over, and wondered what could be the matter with me. On arriving at Coimbra, I was sent to the house of a padre, the clergyman of the parish; like most of the padries in these countries, he came of a large family, at least in the female line, and had a very respectable number of nieces, one of whom was so good as to keep house for him. She was rather good looking, and about five and thirty years of age, which in Portugal constitutes a rather elderly lady. I spoke Portuguese tolerably well, and was very civil to boot, and they were both pleased to delight in me. I could tell them the news, and could understand their complaints against the French; and they gave me in return an excellent dinner and a very good bed, in which I slept soundly for the first time for four or five days. On mentioning this in the morning to my kind hostess, she assured me by, a very significant motion of the thumbs, that she knew the cause of evil, and begged to have my little stock of bedding delivered over to her. On coming home from a morning perambulation of the town, she met me in an ecstasy of delight, assured me she had my blankets hanging in the sun all day, and that those fleas which had not hopped out, her servant had duly destroyed. She further assured me they were as long each as her nail. Now as ladies nails are as long in Portugal as they are in England, and sometimes longer, I demurred at this, and she insisted on my assisting her to find one, but there was not one to be discovered by our united researches, so I cannot vouch for their length; but from the appearance of a dead one afterwards produced, I think they may have been half as long.

In due time I crossed the country, narrowly escaped being drowned in the

Guadiana at Jurumenha, and joined the fourth division of infantry near Olivença. I arrived just after sunset, on the flank of the troops, without knowing more than one individual of the whole. The General commanding was a mile off, at a house I had no chance of finding; and, by one of the odd arrangents of the service, the medical staff officer is the only one to whom custom gives no claim on his hospitality, the other staff-officers all living with him. It rained in torrents, with little hope of its termination; there was nothing to be done but to dismount, place one's back against a tree to which the horses were tied, and await patiently dinnerless the approach of another day. I can assure you I have passed pleasanter evenings. Your progenitors, who snored all night by the sides of your mothers or grandmothers, and growled all the mornings the tax-gatherer paid them a visit, had no idea of the comforts we often enjoyed; and no one who had seen my condition in the morning would have pointed me out as the man who, in a short few days, was in the field of battle of Albuhera, to be the arbiter of the lives and limbs of hundreds of his fellow-creatures. The history of that fight has not yet been correctly given: those who know, do not tell the whole truth; and those who do not know, cannot tell it. I found myself the chief of my own arm on that memorable day, the hardest-fought action of the whole war, simply because I was the senior and the junior of my rank. The surgical history of this battle I will some day tell you, and if no one else will, the military history also; suffice it to say, it satisfied me that an injury from the wind of a cannon-ball was nonsense. In the middle of the contest I dismounted, and had just placed my bridle in the hand of the orderly who led the King's mule, when a cannon-shot passed between his head and mine. I could not help asking him if his head was on, nor of laughing outright, when, with the most soldier-like gravity, he wheeled to the front facing me, touched his cap, and hoped also my head was safe. At the battle of Salamanca, the fourth division, commanded by Sir Lowry Cole, found itself, as usual, under the heaviest fire of the enemy. The troops were ordered to lie down under the fire of twelve heavy guns, to which we had only six light ones to reply, and I halted a little in the rear to make my arrangements. As it was plain we were in for a good pelting, the General sent his aide-de-camp, Captain Roverea, to ascertain where I had fixed the field hospital, that the wounded might be directed upon it. I was at this moment going to the front, and saw my friend Roverea approaching, when my horse stopped and ducked, a sort of gambol I did not think he was warranted to make from the quantity of corn he had eaten. This motion was explained in a moment; a twelve-pound shot, which he had seen, but which I had not, plunged into the loose ploughed field a few feet before him, covered us both with dirt, and hopped calmly, but irresistibly, over my shoulder. Roverea was so white in the face that I thought he must be wounded; he said no he was not, and eagerly inquired if I had seen that shot pass. I said I had, and nearly felt it too. "Well," said he, "it nearly took my nose off." It was impossible to resist laughing at this, for my poor friend, although a most excellent, honourable, and upright man, was certainly not handsome; he was short, with a large face, having high cheek-bones, and as small a proportion of nose as was ever allotted to man, so that in profile but very little of it was to be seen. I could not for the life of me help saying, "My dear Roverea, it might have taken off your head, but I will be hanged if it could have taken off your nose." Not all the sal-volatile in the army could have brought the blood more quickly into his face, for he was very tenacious on this point, and he very indignantly replied, "Sir, you are the only man that would have dared to make such a remark." He had been shot in the head at Albuhera; his skull had been fractured, and when delirious, he had thrown himself out of bed, and thought he owed his life to my kindness. He was, therefore, soon pacified, and willingly forgave my joke. He fell honourably, and for his rank gloriously, shot through the same head on the heights in front of Pampeluna.

After the battle I found myself without conveyance, without stores except those that the panniers of the regimental surgeons contained, and encumbered with 3000 wounded in the village of Valverde. The doctors all worked as no men ever worked before, the toil was incessant, we thought ourselves happy in the improvement of many around us, and that our reward would follow in the approbation of the higher authorities, when lo ! to our astonishment, comes a letter from the Adjutant-General, through the Deputy-Inspector of Hospitals, at Elvas, informing him that he had been made acquainted by an officer deserving credit, of the neglect, &c., with which the wounded had been treated ; of his great disapprobation, &c. You may conceive our anger, but that is not the way to meet an attack of this sort ; when a man errs on the wrong side of truth, the only way to settle the matter is to convict him. I therefore read the letter to the commandant, the late Sir Aretas Young, and to all the wounded officers, and then desired them to tell the Adjutant-General the truth : this I forwarded with a request that the person who was now shown to be a villifier might be brought to justice ; but no, the Adjutant-General was pleased to express in reply, his happiness at finding he had been mistaken as to the wounded at Valverde, but thought the word villifier was too strong. I entreated the Deputy Inspector to go and insist upon an apology from the officer or a reprimand for him from the Adjutant-General, but it was of no use, the deputy was a worthy man but who would as soon have faced the devil as an Adjutant-General, and he gravely wrote me word back, we had got remarkably well out of the scrape and to be quiet. I was not at all contented, but I could not move him. He is long since dead, and I believe with the most profound dread of both these potentates. Well, gentlemen, the matter ended thus : the English papers were full of our valour ; our courage, and our difficulties were the theme of every tongue, the humanity men were even satisfied ; the General and staff-officers obtained stars and ribbons, the officers commanding regiments, whether in or out of action, received medals, many of them were promoted, the regiments inscribed Albuhera on their colours in letters of gold ; some few persons of inferior note who had disappeared and had been reported dead, returned to life ; the poor doctors alone got nothing. Do you wonder now, gentlemen, that a staff-surgeon even, might prefer a comfortable bed and a good dinner at Santarem or Abrantes, at Portalegre or at Elvas, to the field of Albuhera or the trenches of Badajos. I for one however, got something. I carried away with me a little reputation, owing more to circumstances than to any merit of my own, which with a little more gained in different ways, has enabled me, careless of the past, thus freely and laughingly to address you, for

Ridentem dicere verum
Quid vetat?

"*Mais revenons á nos moutons*," an idiomatic expression often used in France, which signifies when duly translated into English, stick to your proper business.

*(To be continued.)*

